# A chromosome-level genome assembly for the beach-spawning California grunion, *Leuresthes tenuis*

**DOI:** 10.1093/jhered/esag012

**Published:** 2026-02-02

**Authors:** Mira Abrecht, Karen L M Martin, Hayden P Speck, Merly Escalona, Ruta Sahasrabudhe, Mohan P A Marimuthu, Oanh Nguyen, Noravit Chumchim, Eric Beraut, Samuel Sacco, William Seligmann, Colin W Fairbairn, Courtney Miller, Elizabeth Heath-Heckman, Erin Toffelmier, H Bradley Shaffer, David K Jacobs

**Affiliations:** Department of Ecology and Evolutionary Biology, University of California, Los Angeles, CA, United States; Department of Biology, Pepperdine University, Malibu, CA, United States; Department of Ecology and Evolutionary Biology, University of California, Los Angeles, CA, United States; Department of Biomolecular Engineering, University of California, Santa Cruz, CA, United States; DNA Technologies and Expression Analysis Core Laboratory, Genome Center, University of California, Davis, CA, United States; DNA Technologies and Expression Analysis Core Laboratory, Genome Center, University of California, Davis, CA, United States; DNA Technologies and Expression Analysis Core Laboratory, Genome Center, University of California, Davis, CA, United States; DNA Technologies and Expression Analysis Core Laboratory, Genome Center, University of California, Davis, CA, United States; Department of Ecology and Evolutionary Biology, University of California, Santa Cruz, CA, United States; Department of Ecology and Evolutionary Biology, University of California, Santa Cruz, CA, United States; Department of Ecology and Evolutionary Biology, University of California, Santa Cruz, CA, United States; Department of Ecology and Evolutionary Biology, University of California, Santa Cruz, CA, United States; Department of Ecology and Evolutionary Biology, University of California, Los Angeles, CA, United States; Department of Integrative Biology, Michigan State University, East Lansing, MI, United States; Department of Microbiology, Genetics, and Immunology, Michigan State University, East Lansing, MI, United States; Department of Ecology and Evolutionary Biology, University of California, Los Angeles, CA, United States; La Kretz Center for California Conservation Science, Institute for Environment and Sustainability, University of California, Los Angeles, CA, United States; Department of Ecology and Evolutionary Biology, University of California, Los Angeles, CA, United States; La Kretz Center for California Conservation Science, Institute for Environment and Sustainability, University of California, Los Angeles, CA, United States; Department of Ecology and Evolutionary Biology, University of California, Los Angeles, CA, United States

**Keywords:** Atherinopsidae, beach, California conservation genomics project, CCGP, conservation genetics, endemic, genomics

## Abstract

We generated the first chromosome-level genome assembly for California grunion, *Leuresthes tenuis*, using PacBio HiFi long reads and Omni-C chromatin-proximity sequencing, yielding a 0.917 Gb genome with a scaffold N50 of 35 Mb and a BUSCO completeness score of 99.37. This beach-spawning marine silverside is the target of a unique recreational hand-grab fishery during its nocturnal spawning runs. Regulation of this fishery, initiated in 1927, remained unchanged from 1949 to 2022, when recent data suggesting a stock decrease led California Department of Fish and Wildlife to reduce the fishing season length. California grunion are endemic to the coast of California and northern Baja California, but within the last two decades the northern limit of spawning has expanded roughly 470 km from Point Conception to north of San Francisco Bay. This genome will facilitate studies addressing the temporal and spatial genetic stock structure, and recent range expansion, of this unique charismatic native species and will also allow assessment of genetic responses to present and future environmental challenges such as changing temperature, and pollution as well as the impacts of harvest and effects of management.

## Introduction

**Fig. 1:**
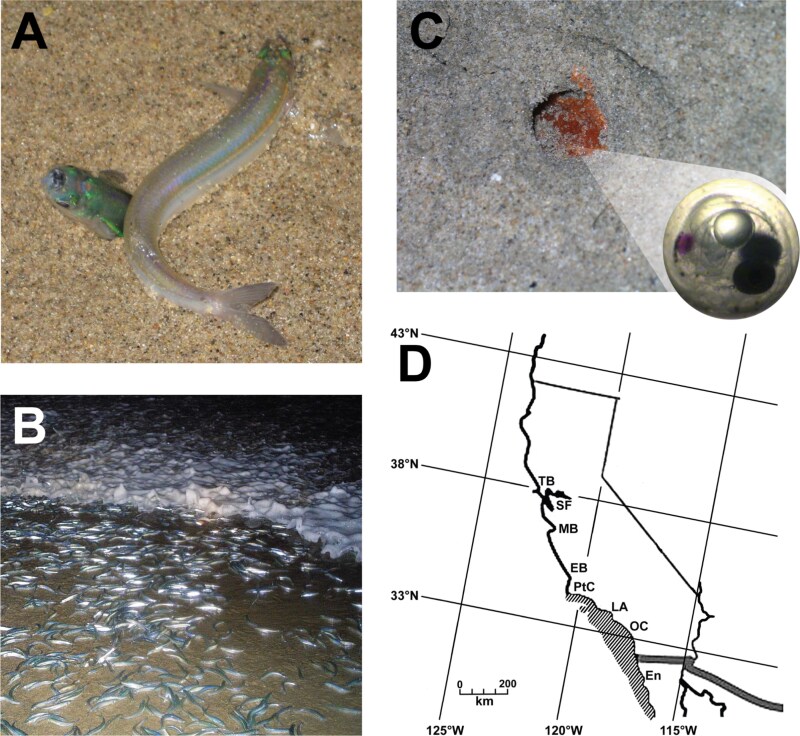
California grunion (*Leuresthes tenuis*) spawning behavior and geographic range. A) Male (on surface) and female (buried tail first, head out) spawning grunion during a grunion run (D Martin). B) Mass onshore spawning of grunion at night in Los Angeles County, CA (W Hootkins). C) Grunion eggs incubate in the high intertidal zone, fully out of water under a few centimeters of sand (C Carreon). Inset shows developing grunion embryo in egg, after yolk has been consumed (K Martin). D) Habitat range in California for California grunion, taken from Johnson et al. 2009. Tomales (TB), San Francisco (SF), Monterey (MB), and Estero Bays (EB) represent discontinuous spawning locations. South of point conception (PtC), range is continuous through Ensenada, Mexico (En), including sites like Surfrider Beach in Malibu (LA) and Doheny Beach in Orange County (OC).

California grunion (*Leuresthes tenuis*), a marine silverside (order Atheriniformes), is widely known for its remarkable spawning behavior. In spring and summer, following the full and new moons which yield the highest semilunar tides, individual grunion aggregate offshore at night and then ride waves onto sandy beaches to spawn. Remaining onshore as a wave recedes, females dig tail-first into the wet sand to lay clutches containing up to 3,000 eggs (Martin 2015). Males curl around the females on the sand surface (Fig. 1A) and provide sperm for multiple females during a run using a special muscle on the urogenital papilla (Aryafar et al. 2019). Spawning runs range from a few to many thousands of fish on shore at one time (Fig. 1B) (Martin et al. 2020), and multiple paternity of a single female’s clutch is common (Byrne and Avise 2009). These mass spawning events often happen on beaches within major Southern California urban centers, and are regularly monitored by dedicated community scientists, and witnessed by thousands of people each year (Martin et al. 2021; Martin and Studer 2022).

The California grunion’s demersal, nonadherent eggs (Fig. 1C) incubate in damp sand above the waterline (Martin et al. 2004; Martin et al. 2009; Moravek et al. 2011) and are ready to hatch approximately 10 days after fertilization. However, hatching requires an environmental trigger: the rush of water washing them back out to sea with the subsequent higher tides of the next new or full moon (Griem and Martin 2000). If this first fortnightly semilunar tide does not wash out the eggs, embryos suspend development (Martin et al. 2011b) remaining metabolically active and ready to hatch (Martin and Podrabsky 2017) for up to 40 days post fertilization depending on the temperature, allowing them to respond to subsequent high tide/wave events (Smyder and Martin 2002; Martin et al. 2011a).

Historically, the California grunion ranged from Punto Abreojos, Baja California (26.71°N, 113.58°W) to Point Conception, California (34.45°N, 34.47°W). Prior isozyme, allozyme, and genetic analysis indicate a slight tendency toward isolation by distance with no distinct populations (Crabtree 1983; Gaida et al. 2003; Byrne et al. 2013). Grunion spawn occasionally in Monterey Bay (Phillips 1943), though fish from this area are not genetically distinct from southern spawners, based on early genetic evidence (Bernardi et al. 2003; Byrne et al. 2013). Since 2002, however, the species’ northern range limit has expanded to Sand Point at the mouth of Tomales Bay north of San Francisco and into San Francisco Bay (Roberts et al. 2007), roughly 470 km upcoast from Point Conception (Fig. 1D). Grunion spawning in San Francisco Bay are genetically indistinguishable from Southern California samples based on microsatellite and mitochondrial sequence analyses, suggesting recent or recurrent colonization (Johnson et al. 2009). However, morphologically, these northern grunion do not grow as large as those from the historic southern portion of the range, constraining egg production (Martin et al. 2013). Whether these northern extralimital populations experience distinct selection from those in Southern California may potentially be addressed with our new genome.

Because of their nearshore and onshore life stages, California grunion are exposed to numerous human impacts. The species has been studied as a model for responses to stressors, such as pesticides (Goodman et al. 1985), salinity (Matsumoto and Martin 2008; Corona and Kültz 2024), microplastics (Uy and Johnson 2022), beach sand replenishment (Lawrenz-Miller 1991; Martin and Adams 2020), beach grooming (Martin et al. 2006), oil spills (Winkler et al. 1983; Hose and Puffer 1984; Gellert et al. 1994), and ocean acidification (Tasoff and Johnson 2019; Johnson 2022; Siegfried and Johnson 2023a, 2023b). Spawning adults are also known to avoid elevated artificial light at night, suggesting another potential anthropogenic impact (Simons et al. 2021).

Here we present a chromosome-level genome assembly for California grunion as part of the California Conservation Genomics Project (CCGP: Shaffer et al. 2022), an effort to provide whole-genome sequencing data for over 250 native California species (Fiedler et al. 2022; Toffelmier et al. 2022). This resource will enable genomic interrogation of demographic change over space and time, genes involved in their unique life history, novel aspects of natural selection, and stress response in California grunion. Beyond California, this genome assembly is a major addition to available genetic data for New World silversides (Atherinopsidae), which are undersampled genomically relative to the rest of the silverside order.

## Methods

### Biological materials

Tissues were dissected on-site from an adult male *L. tenuis* collected by hand during a spawning run at Surfrider Beach in Malibu, Los Angeles County, California (34.036017°N, 118.678217°W) on 30 June 2018 by Martin, Jacobs, and Heath-Heckman under California Scientific Collecting Permit S-21082006-21099-001 (to K.M.), placed on dry ice, and transferred to a −80°C freezer at UCLA prior to shipment for sequencing.

### High molecular-weight genomic DNA isolation

High molecular weight genomic DNA (gDNA) was extracted from 22 mg of muscle tissue using the Nanobind Tissue Big DNA kit following the manufacturer’s instructions (Pacific Biosciences—PacBio, Menlo Park, CA). DNA purity was estimated at absorbance ratios 260/280 = 1.86 and 260/230 = 2.30 by a NanoDrop ND-1000 spectrophotometer (ThermoFisher, Scientific, Waltham, MA) while DNA yield was quantified at 9.6 μg using a QuantiFluor ONE dsDNA Dye assay (Promega, Madison, WI). Assessment of the resulting fragment size distribution using the Femto Pulse Genomic DNA 165 kb assay (Agilent, Santa Clara, CA) indicated that 84% of extracted DNA fragments were 10 kb or longer.

### HiFi library preparation and sequencing

A HiFi SMRTbell library was constructed using the SMRTbell Express Template Prep Kit v2.0 (PacBio, Menlo Park, CA) according to the manufacturer’s instructions. gDNA was sheared to a target size distribution of 15 to 18 kb using Diagenode’s Megaruptor 3 system (Diagenode, Belgium) and concentrated using AMPure PB beads at a 0.45× ratio (PacBio, Menlo Park, CA). gDNA was further processed to remove single-strand overhangs at 37°C for 15 min, repair DNA damage at 37°C for 30 min, repair ends, and add A-tails at 20°C for 10 min followed by 65°C for 30 min, and ligate overhang adapters v3 at 20°C for 60 min. The resulting SMRTbell library was purified and concentrated using AMPure PB beads at a 1× ratio for 30 min preceding and following nuclease treatment. Size selection for fragments greater than 7 to 9 kb via the HippinHT system (Sage Science, Beverley, MA) yielded a final HiFi SMRTbell library 15 to 20 kb in size. Sequencing was performed at UC Davis DNA Technologies Core (Davis, CA) on a PacBio Sequel IIe sequencer using one 8 M SMRT cell, Sequel IIe sequencing chemistry 2.0, and 30-h movie (PacBio, Menlo Park, CA).

### Omni-C library preparation and sequencing

An Omni-C library was prepared using the Dovetail Omni-C Kit (Dovetail Genomics, Scotts Valley, CA) according to the manufacturer’s protocol with slight modifications. Fin tissue was ground with a mortar and pestle while cooled with liquid nitrogen. Subsequently, chromatin was fixed with disuccinimidyl glutarate and formaldehyde. Debris was removed from the suspended chromatin solution with 100 μm and 40 μm cell strainers. Fixed chromatin was digested using a 5-fold dilution of DNase I. After digestion, cells were lysed with sodium dodecyl sulfate, and chromatin fragments were bound to chromatin capture beads. Chromatin ends were repaired and ligated to a biotinylated bridge adapter, followed by proximity ligation of adapter-containing ends. After proximity ligation, crosslinks were reversed, and the DNA was purified from proteins. Purified DNA was treated to remove biotin that was not internal to ligated fragments. An NGS library was generated using an NEB Ultra II DNA Library Prep kit (New England Biolabs, Ipswich, MA) with an Illumina compatible y-adaptor. Biotin-containing fragments were then captured using streptavidin beads. The post capture product was split into two replicates prior to PCR enrichment to preserve library complexity, with each replicate receiving unique dual indices. The library was sequenced at Vincent J. Coates Genomics Sequencing Lab (Berkeley, CA) on an Illumina NovaSeq platform (Illumina, CA) to generate approximately 100 million 2 × 150 bp read pairs per gigabase of genome length.

### RNA isolation, library preparation, and sequencing

Liver and brain tissue from three individual grunion were processed for RNA extraction using TRIzol Reagent (Invitrogen, Carlsbad, CA) followed by DNA removal with the Turbo DNAse kit (Invitrogen, Carlsbad, CA). The resultant RNA was inserted into sequencing libraries using the KAPA RNA HyperPrep Kit (Roche, Switzerland), following the manufacturer’s protocols. RNA quality was measured via Tapestation (Agilent, Santa Clara, CA) before library preparation. RNA libraries were sequenced with 150-bp paired-end reads on the Illumina HiSeq 2,500 system (San Diego, CA) at the UCLA Broad Stem Cell Research Center.

### Nuclear genome assembly

Genome assembly followed the CCGP assembly pipeline Version 5.0 (Table 1; https://github.com/ccgproject/ccgp_assembly). Remnant adapter sequences were removed from the PacBio HiFi sequencing dataset using HiFiAdapterFilt (Sim et al. 2022). Two assemblies, one per haplotype, were generated from an initial diploid phased assembly created using HiFiasm (Cheng et al. 2022) in HiC mode with the filtered PacBio HiFi reads and the Omni-C short reads. Omni-C data were aligned to each assembly following the Arima Genomics Mapping Pipeline (https://github.com/ArimaGenomics/mapping_pipeline). Following this, assemblies were scaffolded with SALSA (Ghurye et al. 2017; Ghurye et al. 2019).

**Table 1:** Tools and nondefault parameters used in CCGP assembly pipeline version 5.0. Software citations are listed in the text.

Assembly step	Software and nondefault options	Version
**Initial assembly**		
Filtering PacBio HiFi adapters	HiFiAdapterFilt	Commit 64d1c7b
K-mer counting	Meryl (k = 21)	1
Estimation of genome size and heterozygosity	GenomeScope	2
De novo *assembly (contiging)*	HiFiasm (Hi-C Mode, −primary, output hic.hap1.p_ctg, hic.hap2.p_ctg)	0.16.1-r375
**Scaffolding**		
Omni-C data alignment	Arima Genomics Mapping Pipeline	Commit 2e74ea4
Arima Genomics Mapping Pipeline (AGMP)	BWA-MEM	0.7.17-r1188
	samtools	1.11
	filter_five_end.pl (AGMP)	Commit 2e74ea4
	two_read_bam_combiner.pl (AGMP)	Commit 2e74ea4
	picard	2.27.5
Omni-C Scaffolding	SALSA (-DNASE, -i 20, -p yes)	2
**Omni-C contact map generation**		
Short-read alignment	BWA-MEM (-5SP)	0.7.17-r1188
SAM/BAM processing	samtools	1.11
SAM/BAM filtering	pairtools	0.3.0
Pairs indexing	pairix	0.3.7
Matrix generation	cooler	0.8.10
Matrix balancing	hicExplorer (hicCorrectmatrix correct --filterThreshold -2 4)	3.6
Contact map visualization	HiGlass	2.1.11
	PretextMap	0.1.4
	PretextView	0.1.5
	PretextSnapshot	0.0.3
Manual curation tools	Rapid curation pipeline (Wellcome Trust Sanger Institute, Genome Reference Informatics Team)	Commit 7acf220c
**Genome quality assessment**		
Basic assembly metrics	QUAST (--est-ref-size)	5.0.2
Assembly completeness	BUSCO (--m geno, -l actinopterygii)	5.0.0
	Merqury	2020-01-29
**Contamination screening**		
Local alignment tool	BLAST+ (-db nt, -outfmt ‘6 qseqid staxids bitscore std’, -max_target_seqs 1, -max_hsps 1, -evalue 1e-25)	2.15
General contamination screening	BlobToolKit (HiFi coverage, BUSCO = actinopterygii, NCBI Taxa ID = 355 514)	2.3.3

Assemblies for both haplotypes were manually curated by iteratively generating and analyzing their corresponding Omni-C contact maps. Contact maps were generated by aligning Omni-C data with BWA-MEM (Li 2013), then identifying ligation junctions and Omni-C pairs (Lee et al. 2022) using pairtools (Open2C et al. 2024). Multi-resolution Omni-C matrices were then generated with cooler (Abdennur and Mirny 2020) and balanced with hicExplorer (Ramírez et al. 2018). Contact matrices were visualized using HiGlass (Kerpedjiev et al. 2018) and PretextSuite (https://github.com/wtsi-hpag/PretextView;  https://github.com/wtsi-hpag/PretextMap;  https://github.com/wtsi-hpag/PretextSnapshot) to identify misassemblies and misjoins, which were addressed using the Rapid Curation pipeline from the Wellcome Trust Sanger Institute, Genome Reference Informatics Team (https://gitlab.com/wtsi-grit/rapid-curation). Remaining gaps, including joins generated during scaffolding and/or curation, were closed using the PacBio HiFi reads and YAGCloser (https://github.com/merlyescalona/yagcloser). Finally, contamination was screened using the BlobToolKit Framework (Challis et al. 2020).

### Genome quality assessment

K-mer counts were generated from adapter-filtered PacBio HiFi reads using meryl (Rhie et al. 2020), then used to estimate genome size, heterozygosity, and repeat content in GenomeScope2.0 (Ranallo-Benavidez et al. 2020). General contiguity metrics were generated via QUAST (Gurevich et al. 2013), while genome quality and functional completeness were evaluated using BUSCO (Manni et al. 2021) with the Actinopterygii ortholog database (actinopterygii_odb10), which contains 3,640 genes. Assessment of base level accuracy (quality value, QV) and k-mer completeness was performed using the merqury (Rhie et al. 2020) and the previously generated meryl database. Assembly accuracy was further evaluated via BUSCO gene set frameshift analysis following Korlach et al. 2017. Following this, the phased block size was measured based on the size of the contigs generated by HiFiasm on HiC mode.

Quality metrics for the first haplotype assembly are reported following the nomenclature established by Rhie et al. 2021: for genome quality code x.y.P.Q.C, x is log_10_[contig NG50], y is log_10_[scaffold NG50], P is log_10_ [phased block NG50], Q is Phred base accuracy QV, and C is the percentage of the genome represented by the first n scaffolds following a karyotype of 2n = 48 as estimated from the ancestral species number of chromosomes (Genome on a Tree, GoaT; tax_name (*L. tenuis*); Challis et al. 2023).

### Genome annotation

The assembly for one haplotype (fLeuTen1.0.hap1) was annotated through the NCBI Eukaryotic Genome Annotation Pipeline (Thibaud-Nissen et al. 2013). In brief, this pipeline used alignments to RNA-seq and protein data from related taxa to inform models of gene prediction and subsequent genome annotation. A comprehensive list of RNA-seq and protein data used in this annotation is available on the NCBI Annotation Release page for the assembly (NCBI *L. tenuis* Annotation Release GCF_036924035.1-RS_2025_03: https://www.ncbi.nlm.nih.gov/refseq/annotation_euk/Leuresthes_tenuis/GCF_036924035.1-RS_2025_03/).

### Genome assembly comparison within Atheriniformes

We searched GenBank for atheriniform genomes (query: https://www.ncbi.nlm.nih.gov/datasets/genome/?taxon=461499) and selected a single-genome assembly per species based on its designation as a “reference” or “representative genome” by NCBI. The genome assemblies were downloaded using NCBI datasets (O’Leary et al. 2024), and for each assembly, we generated contiguity metrics using QUAST and calculated functional completeness using BUSCO in conjunction with the Actinopterygii ortholog database (actinopterygii_odb10). Generic and species diversities were determined using FishBase (Froese and Pauly 2025).

## Results

Genome assembly and annotation data were deposited on NCBI GenBank, while RNA-seq data were deposited on NCBI Short Read Archive (SRA). Accession numbers, as well as sequencing and assembly statistics, are presented in Table 2.

**Table 2:** Accession numbers, sequencing, and assembly statistics.

**Bio-projects and vouchers**					
CCGP NCBI Bio-project	PRJNA720569 http://www.ncbi.nlm.nih.gov/bioproject/PRJNA720569				
* Leuresthes* NCBI Bio-project	PRJNA986188 https://www.ncbi.nlm.nih.gov/bioproject/PRJNA986188				
NCBI Bio-sample	SAMN36908943 https://www.ncbi.nlm.nih.gov/biosample/SAMN36908943				
Specimen ID number	DKJ/LTE-2018-01-01				
SRA RNA-seq Bio-project	PRJNA804529 https://www.ncbi.nlm.nih.gov/bioproject/PRJNA804529				
NCBI genome accessions	**Haplotype 1**			**Haplotype 2**	
Assembly	JAVGWV000000000			JAVGWW000000000	
Genome sequences	GCA_036924035.1			GCA_036924055.1	
Annotation release	GCF_036924035.1-RS_2025_03 https://www.ncbi.nlm.nih.gov/refseq/annotation_euk/Leuresthes_tenuis/GCF_036924035.1-RS_2025_03/				
**Genome sequence**					
PacBio HiFi long read runs	1 PACBIO_SMRT (Sequel II) run: 2.4 M spots, 32.3G bases, 19.5Gb				
PacBio HiFi NCBI SRA accession	SRX25151346 https://www.ncbi.nlm.nih.gov/sra/SRX25151346				
OmniC Illumina sequencing	2 Illumina NovaSeq 6000 runs: 135.8 M spots, 41G bases, 13.8 Gb				
OmniC Illumina NCBI SRA accession	SRX25151347-8 https://www.ncbi.nlm.nih.gov/sra/SRX25151347				
**Genome assembly quality metrics**					
Assembly identifier (quality code^a^)	fLeuTen1 (7.7.P7.Q65.C93)				
HiFi Read coverage^b^	39.19X				
	**Haplotype 1**			**Haplotype 2**	
Number of contigs	518			662	
Contig N50	9,667,060 bp			9,883,555 bp	
Contig NG50^b^	11,642,777 bp			11,322,310 bp	
Longest contig	26,413,092 bp			28,543,195 bp	
Number of scaffolds	295			440	
Scaffold N50	35,077,053 bp			35,848,745 bp	
Scaffold NG50^b^	36,527,599 bp			36,252,266 bp	
Largest scaffold	44,676,152 bp			46,791,244 bp	
Size of final assembly	917,098,913 bp			899,536,685 bp	
Phased block NG50^b^	11,250,983 bp			11,642,777 bp	
Gaps per Gbp (number of gaps)	243 (233)			247 (222)	
Indel QV (frame shift analysis)	Q 49.1033			Q 48.3584	
Base pair QV	Q 65.6916			Q 65.6916	
	Full assembly: Q 65.7304				
k-mer completeness	89.4849%			89.3801%	
	Full assembly: 99.4303%				
BUSCO completeness (Actinopterygii)^c^ n=3640	**C**	**S**	**D**	**F**	**M**
H1^d^	99.37%	99.01%	0.36%	0.44%	0.19%
H2^d^	99.37%	99.12%	0.25%	0.49%	0.14%

^a^Assembly quality code notation x.y.P.Q.C (derived from Rhie et al. 2021): x = log10[contig NG50]; y = log10[scaffold NG50]; P = log10 [phased block NG50]; Q = Phred base accuracy QV; C = % genome represented by the first “n” scaffolds, following a karyotype of 2n = 48 for this species, estimated as a mode from ancestral species number of chromosomes (Genome on a Tree, GoaT; tax_name (*Leuresthes tenuis*); Challis et al. 2023). Quality code for assembly is denoted by haplotype assembly fLeuTen1.0.hap1.

^b^Read coverage and NG50 statistics have been calculated based on the estimated genome size of 0.824 Gb.

^c^BUSCO Scores. Complete BUSCOs (C). Complete and single-copy BUSCOs (S). Complete and duplicated BUSCOs (D). Fragmented BUSCOs (F). Missing BUSCOs (M).

^d^Assembly values are for haplotype one (H1) and haplotype two (H2).

### Sequencing data

**Fig. 2:**
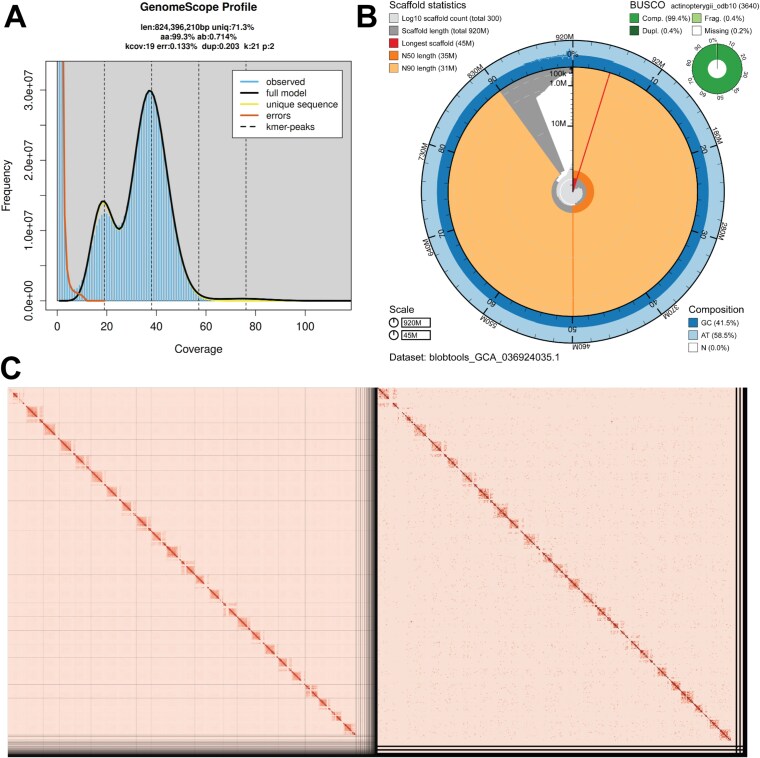
Graphical summary of sequencing and assembly statistics. A) K-mer spectrum generated from PacBio HiFi data using GenomeScope2.0. Actual (observed) and estimated (full model) k-mer profiles are plotted alongside unique sequences, errors, and k-mer peaks. The k-mer profile is bimodal, suggestive of significant heterozygosity consistent with a large potentially panmictic population. Sequencing statistics include estimated genome length (len), percentage of genome that is unique (uniq), overall rate of homozygosity (aa) and heterozygosity (ab), average k-mer coverage for heterozygous bases (kcov), error rate (err), average read duplication rate (dup), k-mer size (k), and ploidy (p). B) Snail plot generated for haplotype one (fLeuTen1.0.hap1) assembly using BlobToolKit. The full size of the assembly is represented by the larger plot circle, with scaffold N50 and N90 lengths indicated by dark and light orange arcs, respectively. The size of the largest scaffold is marked by a red radial line, while all other scaffolds, drawn in dark grey, are arranged clockwise and spiral inwards in order of size. Cumulative scaffold count is shown in light grey, with order of magnitude marked with white dashed lines. In this area, the proportion of Ns in the assembly is displayed in white, while mean, maximum, and minimum GC and AT content is displayed in dark blue and light blue, respectively. BUSCO scores for the Actinopterygii gene set are represented as the smaller plot circle, with complete, fragmented, duplicated, and missing BUSCOs drawn in green, light green, dark green, and white, respectively. A similar snail plot for haplotype two (fLeuTen1.0.hap2) can be found in Supplementary Fig. S2. C) Omni-C contact maps generated for haplotype one (left) and haplotype two (right) using PretextSnapshot. Each cell in the contact map corresponds to linkage between two genomic regions, with darker colors representing greater support for this linkage. Scaffolds are separated by black lines, while higher density corresponds to higher levels of fragmentation.

The Omni-C library generated 135.8 million read pairs, while the PacBio HiFi library generated 2.44 million reads. PacBio HiFi sequences yielded ~39× genome coverage with an N50 read length of 13,690 bp, minimum read length of 244 bp, mean read length of 13,231 bp, and maximum read length of 48,351 bp (Supplementary Fig. S1). Based on long read data, GenomeScope 2.0 estimated a genome size of 824.39 Mb, a sequencing error rate of 0.133%, and heterozygosity at 0.714%. The k-mer spectrum shows a bimodal distribution with a major peak at ~38× coverage and a minor peak at ~19× coverage (Fig. 2A).

### Nuclear genome assembly

The final genome assembly of *L. tenuis* (fLeuTen1) consists of two phased haplotypes: haplotype one (fLeuTen1.0.hap1) and haplotype two (fLeuTen1.0.hap2). Both assemblies are similar in length, and are larger than the genome size estimated by Genomescope 2.0, a pattern that has been observed in other taxa (see Pflug et al. 2020, for example).

The haplotype one assembly consists of 295 scaffolds spanning 917.09 Mb with a contig N50 of 9.66 Mb, scaffold N50 of 35.07 Mb, largest contig size of 26.41 Mb, and largest scaffold size of 44.68 Mb. Frameshift indel QV was 49.1, base pair QV was 65.69, k-mer completeness was 89.48%, and BUSCO completeness was 99.73% (Actinopterygii gene set). The haplotype two assembly consists of 440 scaffolds spanning 899.53 Mb with a contig N50 of 9.88 Mb, scaffold N50 of 35.84 Mb, largest contig size of 28.54 Mb, and largest scaffold size of 46.79 Mb. For haplotype two, frameshift indel QV was 48.35, base pair QV was 65.69, k-mer completeness was 89.38%, and BUSCO completeness was 98.7%. During manual curation, 377 joins (169 on haplotype one and 168 on haplotype two) and 12 breaks (5 on haplotype one and 7 on haplotype two) were made, while 33 gaps were closed (20 on haplotype one and 13 on haplotype two). No other contigs were removed or modified.

Omni-C contact maps for haplotype one show highly contiguous assemblies with chromosome-length scaffolds (Fig. 2B; see Supplementary Fig. S2 for haplotype two). Other assembly statistics are represented graphically in Fig. 2C.

### Genome annotation

The final genome annotation included 55,513 genes and pseudogenes, 36,304 mRNAs and 17,050 noncoding RNAs, and 36,386 coding sequences. BUSCO analysis reports a completeness score of 99.3%, with 98.4% of genes presenting as single-copy, 0.9% as duplicated, 0.4% as fragmented, and 0.3% as missing. Additional metrics can be found in Supplementary Table S2.

### Genome assembly comparison across Atheriniformes

Comparison to other assemblies indicates that the California grunion genome is the most complete atheriniform genome as inferred by BUSCO completeness (Table 3), with low percentages of fragmented genes and BUSCO missingness comparable to Argentinian silverside (*Odontesthes bonariensis*) and Boeseman’s rainbowfish (*Melanotaenia boesemani*) genomes. Of the 42 atheriniform genomes available, 24 have BUSCO scores over 90% while several are below 20%. Thus, genome quality should be considered in selecting taxa for comparative work within Atheriniformes. Silverside genomes are relatively small, ranging in ungapped length from 1.2 Gb in the hardhead silverside (*Atherinomorus stipes*) to 0.39 Gb in the Kokas rainbowfish (*Melanotaenia affinis*) for an average of 0.7 Gb (Table 3). Thus, the ungapped length (0.92 Gb) of the California grunion genome is roughly a third larger than average for the order. The grunion-containing subfamily Atherinopsinae, with an average genome size of 0.93 Gb (*n* = 2), may have larger genomes than its sister Menidinae, with an average of 0.55 Gb (*n* = 3). Further sampling is needed to test this hypothesis, and inspection of genome size across atheriniform taxa does not otherwise suggest dramatic patterns in genome size.

**Table 3:** Grunion genome relative to other silverside genomes. This table provides standard comparative metrics for genomes of the order Atheriniformes (silversides and rainbowfish) listed relative to their phylogenetic proximity to the California grunion genome reported here. Generic and species number within groups permits assessment of genome sequencing effort relative to biodiversity (comparable to a table provided for gobies Jacobs et al. 2025). Taxonomic treatment shows family, subfamily, and tribes within the suborders Atherinopsoidei and Atherinoidei (the New World silversides, and Old World silversides and rainbowfishes, respectively) following the phylogeny developed by Campanella et al. 2015. Consequently, “Notocheirinae” is placed within the family Atherinopsidae. All species with genomes are shown. Generic and species diversity of groups lacking genome sequencing are also shown to facilitate discussion of the state of genomics relative to biodiversity. Species and generic diversity numbers are derived from Fishbase (Froese and Pauly 2025). Common names are abbreviated for space as follows: svs(s), silverside(s); hdh, hardyhead; be, blue-eye; rbf(s), rainbowfish(es); ppm, priapiumfish. Abbreviations for assembly level are as follows: CO, contig; SC, scaffold; CH, chromosome. Dashed line separates tribe Atherinopsini from tribe Sorgentini within the New World silversides, jagged line separates subfamily Atherinopsinae from other subfamilies within the new world silversides, and double jagged line separates the New and Old World silverside suborders.

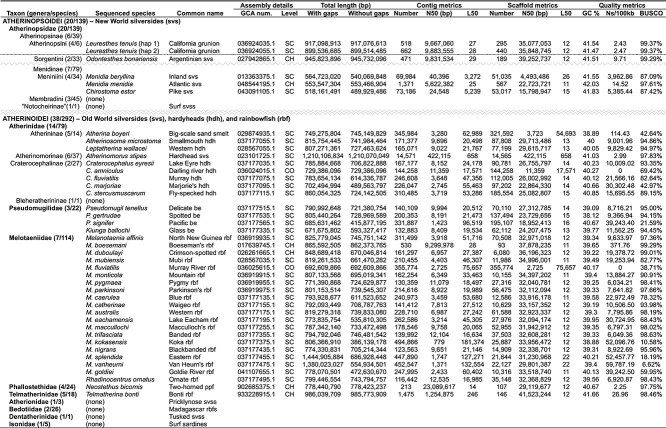

## Discussion

Terrestrial incubation may make California grunion more susceptible to global warming than most other marine fishes (Smyder and Martin 2002; Shelley and Johnson 2022; Moore et al. 2023), and they may be approaching lethal limits for egg incubation in the southern portions of their traditional range. Over the past two decades, surveys of Southern California beaches indicate that breeding numbers have declined (Martin et al. 2020). However, recent northward expansion of the species may indicate a capacity for relocation to novel spawning locations (Martin et al. 2013), although reductions in adult body size, reproductive output, and possibly lifespan appear to characterize these northern extralimital populations.

Genomic comparison of California grunion with other fishes could reveal the underlying genetic controls of hatching readiness via the activation of hatching enzymes (Castro et al. 2021), features of metabolism extending incubation during hatching delay (Thompson and Ortí 2016; Martin and Podrabsky 2017; Thompson et al. 2017), the regulation of the lunar spawning cycle by clock genes and hormones (Reinberg et al. 2016; Chen et al. 2022), and the genetic basis of chorion changes during development (Carter and Dickson 2014). The response of California grunion to natural and human-induced environmental stressors can similarly be examined at the genomic level with the assembly presented here. Other key features of California grunion that can, and should, be interrogated genomically include fluctuating asymmetry in morphological characters in the presence of stressors (Valentine and Soulé 1973; Leary and Allendorf 1989; Parsons 1992), the influence of photoperiod and temperature on sex determination (Brown et al. 2014), the timing of development with respect to environmental signals (Smyder and Martin 2002; Martin et al. 2009; Moravek and Martin 2011), vertebral number (McHugh 1954), and the impacts of pollution and altered salinity (Kirchhoff et al. 1999). Significantly, the relationship of maternal investment to genetics and environment may also now be examined (Schultz 1991; Johnson et al. 2024) to investigate environmental drivers of smaller clutch sizes and egg diameters in San Francisco Bay grunion compared with southern grunion (Moffatt and Thomson 1978).

California grunion genomics may also inform appropriate management actions. With harvest currently being managed by setting dates for the open season, it may be important to identify genetic differences between early- and late-season runs and the molecular mechanisms driving these differences. Additionally, given recent catastrophic impacts on beaches due to fire, understanding local differentiation will ensure that restoration efforts are appropriately sourced if necessary. Lastly, categorizing the demographic and selective impacts associated with the species’ expansion into the San Francisco Bay Area may provide insight into the consequences of further climate change-driven northern expansion of grunion and other taxa along the Pacific Coast.

### Genome assembly comparison within Atheriniformes

Our grunion genome is the only sequenced representative of the North Pacific tribe (Atherinopsini) of the New World silverside subfamily Atherinopsinae (Family Atherinopsidae), which, in addition to *Leuresthes,* includes *Colpichthys, Atherinops*, and *Atherinopsis* (Crabtree 1987). The Gulf grunion, *Leuresthes sardina*, is the California grunion’s sister species and only congener. It should provide interesting functional genomic comparison as it is endemic to the Gulf of Califorina, where it breeds on the beaches during daytime rather than at nightime high tides and is subject to higher summer time temperatures (Carmona et al. 2017). *Colpichthys*, a genus endemic to the Gulf of California, is also of comparative genomic interest due to salinity-related ecological speciation between *C. hubbsi*, endemic to the Colorado Delta, and *C. regis*, which inhabits historically full salinity estuaries through much of the Gulf of California. Admixture between these species has followed loss of freshwater from the Colorado River (Lau and Jacobs 2017). The monotypic genera *Atherinops* (topsmelt silverside) and *Atherinopsis* (jacksmelt silverside) merit further study given their ecological importance in estuarine, kelp, and coastal ecosystems of California (Horn et al. 2006) and their morphological distinction in feeding mechanics relative to *Leuresthes* (Higgins and Horn 2014). The South American sister tribe to Atherinopsini, Sorgentinini (Dyer 1993; Campanella et al. 2015) is genomically represented by *O. bonariensis*, a member of a speciose genus with salinity-driven ecological speciation (Hughes et al. 2020; González-Castro et al. 2022). The sister subfamily to Atherinopsinae, Menidinae, largely occupies the Atlantic coast of the Americas, and is represented by genomes from the genera *Menidia* and *Chirostoma. Menidia* has been studied relative to salinity-associated speciation (Fluker et al. 2011), while *Chirostoma* is a speciose group endemic to the highlands of the central Mexican Plateau (Barbour 1973). The diverse tribe Membradini (within Menidinae) is currently unrepresented by any species with a genome-level assembly.

Suborder Atherinopsoidei (New World silversides) is now represented by five species with genomes out of 139 currently recognized species, whereas their sister taxon Atherinoidei (Old World silversides and rainbowfish) is represented by 35 genomes out of 292 species (Table 3) (Froese and Pauly 2025). We note that application of the term “Old World” broadly to this group is misleading, as only a small number of species, primarily within the widespread genera *Atherina* and *Atheromorus*, are associated with mainland Europe, Asia, and Africa. Instead, much of the diversity is associated with the former east Gondwanan margin–Southeast Asia and the Indo-Pacific. In Australia, silversides (Atherinidae) called hardyheads have diversified in the more southern regions, while several families of rainbowfishes diversified primarily in tropical Australia and New Guinea (Melanotaeniidae and Pseudomugilidae), Sulawesi in Wallacea (Telmatheridae), and Madagascar (Bedotiidae) (Campanella et al. 2015). If further genome sequencing efforts were to focus on poorly sampled diverse groups, sequencing within the speciose genus *Atherinella* within the New World Membradini would be merited, as would sequencing within the speciose and poorly known Madagascar rainbowfishes. The Wallacean rainbowfish group Telmatherinidae and the Philippine and Southeast Asian Phallostethidae also deserve further attention, as do Notocheirinae, Atherionidae, and Isonidae based on their biogeographic and phylogenetic uncertainty within the group (Campanella et al. 2015).

Other marine taxa outside of Atheriniformes may also merit genomic comparison across California (Fiedler et al. 2022). To date, the CCGP has sequenced five fish and five marine invertebrate species on the California coast. Many of the fishes have biological attributes that limit marine dispersal, potentially leading to exceptional genetic subdivision for marine taxa beyond that of the California grunion reported here. The black surf perch (*Embiotoca jacksoni*) is a livebearer lacking larval dispersal (Bernardi et al. 2022b). The wooly sculpin (*Clinocottus analis*) (Wright et al. 2023b), monkeyface prickleback (*Cebidichthys violaceus*) (Wright et al. 2023a), and tidewater goby (*Eucyclogobius newberryi*) (Jacobs et al. 2025) have fertilization, egg laying, and brooding behavior that limits dispersal, while the sequestration of tidewater gobies in closed lagoons during reproduction leads to further isolation (Earl et al. 2010; Swift et al. 2016). Of the fishes, only the California sheephead (*Semicossyphus pulcher*) has external fertilization and marine larvae (Bernardi et al. 2022a), making it likely more dispersive and closer to panmixia than the California grunion. Of the five marine invertebrate genomes sequenced to date by the CCGP, the Pismo clam (*Tivela stultorum*) (Emery et al. 2025), black abalone (*Haliotis cracherodii*) (Orland et al. 2022), and red abalone (*Haliotis rufescens*) (Griffiths et al. 2022) have been impacted by fisheries amongst other factors, while the California mussel (*Mytilus californianus*) (Paggeot et al. 2022) has a long history of human use in the archaeological record (Campbell et al. 2018). The pink sea star (*Pisaster brevispinus*) (DeBiasse et al. 2022) is an ecologically important predator. All these taxa have external fertilization and marine-dispersing larvae, and thus are anticipated to be genetically less subdivided than California grunion. In addition to individual species management, the CCGP is providing resources suitable for comparative study of dispersal process and evolution of marine species along the California Coast.

## Conclusion

Our high-quality genome of the iconic beach-spawning California Grunion will, with ongoing genome resequencing, permit better spatial and temporal management of the watchable wildlife and coastal harvest of this taxon that is so well known as a key element of Southern California beach culture. It should also help clarify how this unique fish species may respond to changing climate. This genome also opens significant opportunities for increased functional, evolutionary, and ecological studies of the globally distributed silversides and rainbowfishes.

## Supplementary Material

Supplementary_Material_esag012

## Data Availability

Data generated for this study are available under NCBI BioProject PRJNA1126710. Raw sequencing data for sample DKJ/LTE-2018-01-01 (NCBI BioSample SAMN36908943) are deposited in the NCBI SRA under SRR29647171 for PacBio HiFi sequencing data, and SRR29647169, SRR29647170 for the Omni-C Illumina sequencing data. GenBank accessions for both primary and alternate assemblies are GCA_036924035.1 and GCA_036924055.1; and for genome sequences JAVGWV000000000.1 and JAVGWW000000000.1. Assembly scripts and other data for the analyses presented can be found at the following GitHub repository: www.github.com/ccgproject/ccgp_assembly.
