# Supplementary material for: A chromosome-level genome assembly for the beach-spawning California grunion, Leuresthes tenuis
Source: J Hered. 2026 Feb 2;117(4):856–67. doi: 10.1093/jhered/esag012 (PMC13326419; doi:10.1093/jhered/esag012)
Supplement: Supplementary_Material_esag012 [file supplementary_material_esag012.docx]

# Supplementary Material

**Supplementary Figure S1. Read length distribution of PacBio HiFi sequencing data.** Mean read length is marked by a red line.


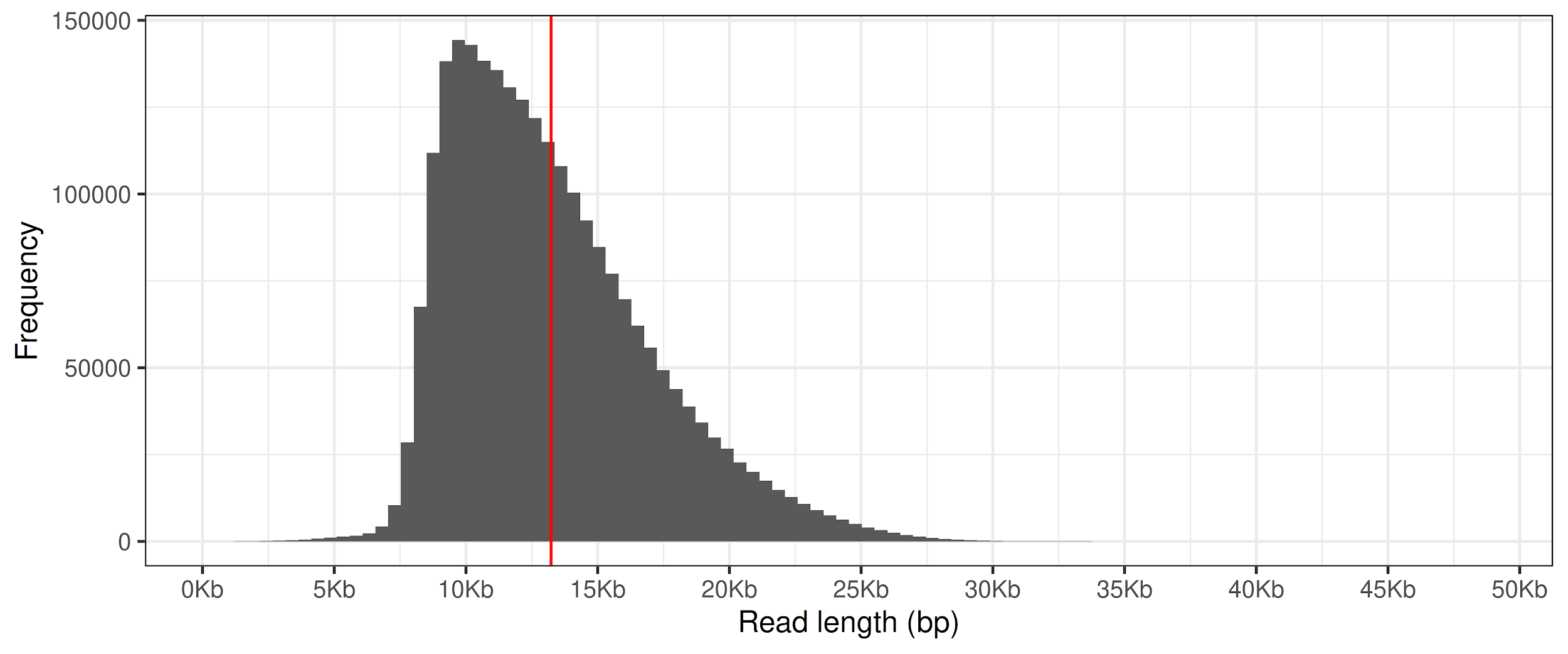


**Supplementary Figure S2. Snail plot generated for haplotype two (fLeuTen1.0.hap2) assembly using BlobToolKit.** The full size of the assembly is represented as the larger plot circle, with scaffold N50 and N90 lengths indicated by dark and light orange arcs, respectively. The size of the largest scaffold is marked by a red radial line, while all other scaffolds, drawn in dark grey, are arranged clockwise and spiral inwards in order of size. Cumulative scaffold count is shown in light grey, with order of magnitude marked with white dashed lines. In this area, the proportion of Ns in the assembly is displayed in white, while mean, maximum, and minimum GC and AT content is displayed in dark blue and light blue, respectively. BUSCO scores for the Actinopterygii gene set are represented as the smaller plot circle, with complete, fragmented, duplicated, and missing BUSCOs drawn in green, light green, dark green, and white, respectively.


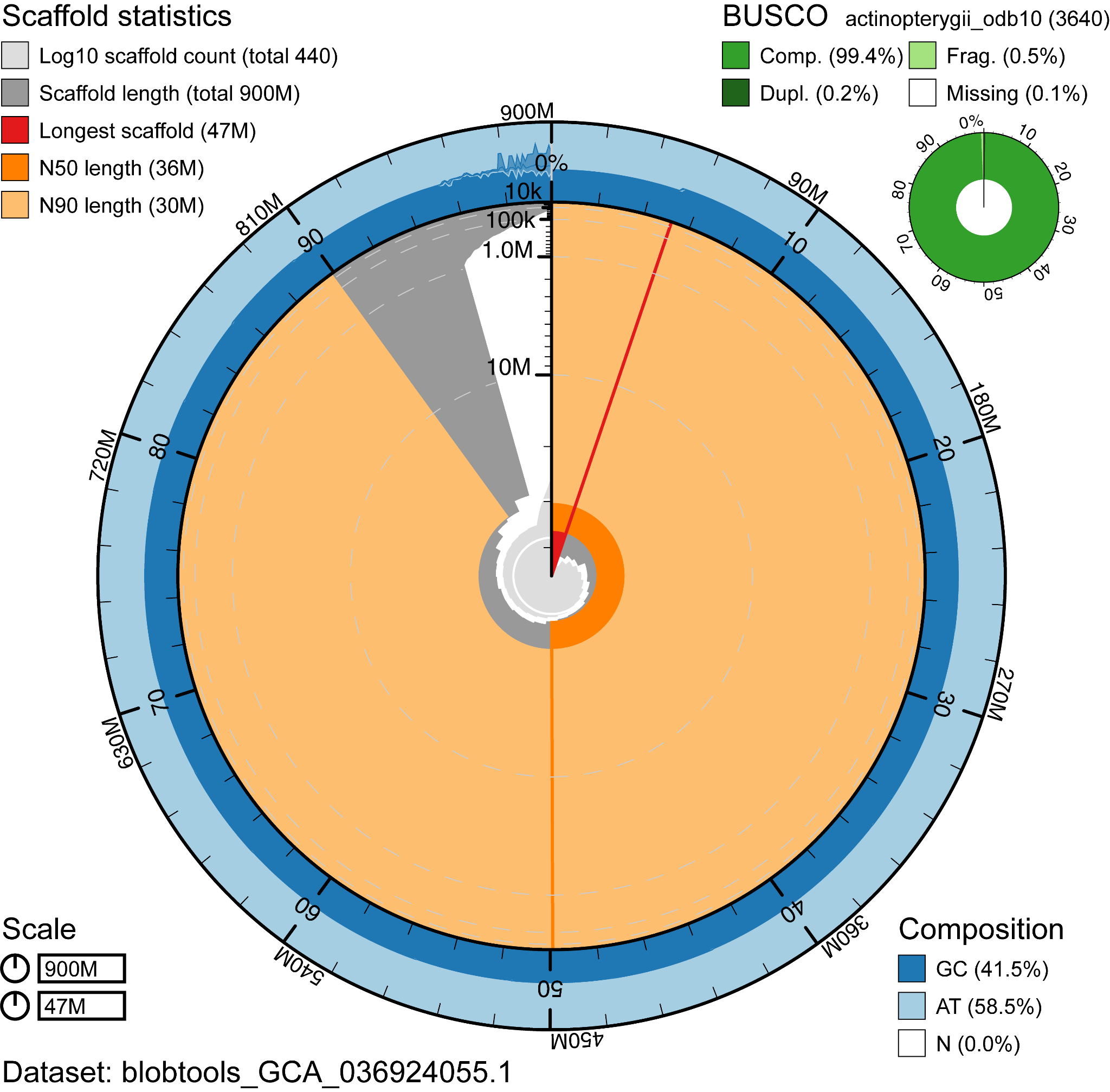


**Supplementary Table S2. Annotation statistics for haplotype one (fLeuTen1.0.hap1) assembly.** Metrics sourced from NCBI Leuresthes tenuis Annotation Release GCF_036924035.1-RS_2025_03 (https://www.ncbi.nlm.nih.gov/refseq/annotation_euk/Leuresthes_tenuis/GCF_036924035.1-RS_2025_03/).

| **Feature counts** | | | | | |
| --- | --- | --- | --- | --- | --- |
| Genes and pseudogenes | 55,513 |  |  |  |  |
| Genes | 38,553 |  |  |  |  |
| Pseudogenes | 16,891 |  |  |  |  |
| All transcripts | 53,534 |  |  |  |  |
| mRNAs | 36,304 |  |  |  |  |
| Non-coding RNA’s | 17,050 |  |  |  |  |
| CDSs | 35,386 |  |  |  |  |
| **Genome annotation quality metrics** | | | | | |
| BUSCO completeness † | **C** | **S** | **D** | **F** | **M** |
| (actinopterygii_odb10, n = 3640) | 99.30% | 98.40% | 0.90% | 0.40% | 0.30% |
| † BUSCO Scores. Complete BUSCOs (C). Complete and single-copy BUSCOs (S). Complete and duplicated BUSCOs (D). Fragmented BUSCOs (F). Missing BUSCOs (M). | | | | | |
